# Neuron-Schwann cell interactions in peripheral nervous system homeostasis, disease, and preclinical treatment

**DOI:** 10.3389/fncel.2023.1248922

**Published:** 2023-10-12

**Authors:** Julia Teixeira Oliveira, Christopher Yanick, Nicolas Wein, Cintia Elisabeth Gomez Limia

**Affiliations:** ^1^Department of Neurology, University of Miami, Miami, FL, United States; ^2^Center for Gene Therapy, Abigail Wexner Research Institute at Nationwide Children’s Hospital, Columbus, OH, United States; ^3^Department of Pediatrics, The Ohio State University, Columbus, OH, United States; ^4^Department of Biomedical Engineering, The Ohio State University, Columbus, OH, United States

**Keywords:** Schwann cells-neurons interaction, regeneration, myelination, pre-clinical trials, repair

## Abstract

Schwann cells (SCs) have a critical role in the peripheral nervous system. These cells are able to support axons during homeostasis and after injury. However, mutations in genes associated with the SCs repair program or myelination result in dysfunctional SCs. Several neuropathies such as Charcot–Marie–Tooth (CMT) disease, diabetic neuropathy and Guillain–Barré syndrome show abnormal SC functions and an impaired regeneration process. Thus, understanding SCs-axon interaction and the nerve environment in the context of homeostasis as well as post-injury and disease onset is necessary. Several neurotrophic factors, cytokines, and regulators of signaling pathways associated with proliferation, survival and regeneration are involved in this process. Preclinical studies have focused on the discovery of therapeutic targets for peripheral neuropathies and injuries. To study the effect of new therapeutic targets, modeling neuropathies and peripheral nerve injuries (PNIs) *in vitro* and *in vivo* are useful tools. Furthermore, several *in vitro* protocols have been designed using SCs and neuron cell lines to evaluate these targets in the regeneration process. SCs lines have been used to generate effective myelinating SCs without success. Alternative options have been investigated using direct conversion from somatic cells to SCs or SCs derived from pluripotent stem cells to generate functional SCs. This review will go over the advantages of these systems and the problems associated with them. In addition, there have been challenges in establishing adequate and reproducible protocols *in vitro* to recapitulate repair SC-neuron interactions observed *in vivo*. So, we also discuss the mechanisms of repair SCs-axon interactions in the context of peripheral neuropathies and nerve injury (PNI) *in vitro* and *in vivo*. Finally, we summarize current preclinical studies evaluating transgenes, drug, and novel compounds with translational potential into clinical studies.

## 1. Introduction

The peripheral nervous system (PNS) is part of the nervous system outside the brain and spinal cord. It plays a key role in providing information to the brain and from the brain towards the organs. Peripheral nerves are surrounded by glial cells called Schwann cells (SCs), which sometimes cover axons with a myelin sheath, and are the major glial cell type in the PNS. They play essential roles in the development, maintenance, function, and regeneration of peripheral nerves (Jessen et al., 2015). Myelinating SCs provide large axons with a myelin sheath, an insulating layer that forms around nerves. This insulation sheath allows the quick and efficient transmission of action potentials along axons. PNS also contains non-myelinating SCs, named Remak cells, a class of SCs that form Remak bundles, which support small-caliber axons during homeostasis and after injury.

**Figure 1:**
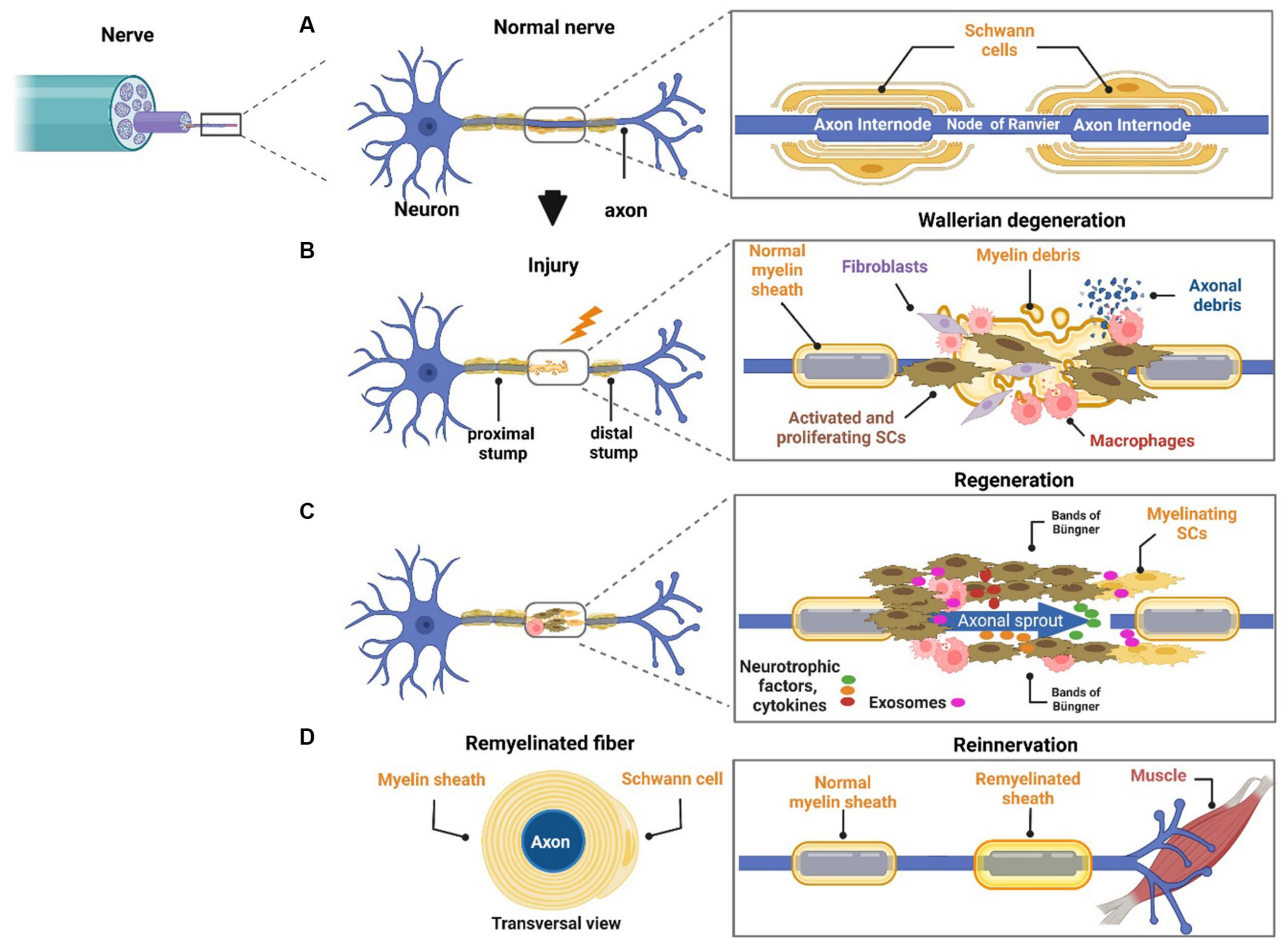
Role of neurons and SCs in regeneration. The peripheral nerve is mainly formed by axons and SCs. SCs have a crucial role in nerve homeostasis and regeneration by supporting axons by several means. SCs myelinate large axons **(A)**. In response to injury, Wallerian degeneration takes place. SCs dedifferentiate, proliferate, and start myelin debris clearance. Macrophages migrate to the local area to take over phagocytosis of myelin and axonal debris and to interact with other cells in the injured microenvironment **(B)**. Differentiated SCs form bands of Büngner and remyelinate the axon. Several neurotrophic factors, cytokines, and exosomes are released from the local cell population interacting with axons. In this sequence, the axon starts outgrowing in an attempt to reinnervate the target organ **(C)**. Successful reinnervation is the final goal of nerve regeneration, but it is seldom achieved because it depends on several factors such as age, severity of injury, and distance from the cell body **(D)**.

Either myelinating or unmyelinating SCs play a very important role during nerve trauma. Such events trigger several orchestrated cellular and molecular events called Wallerian degeneration that led to regeneration of the proximal nerve stump while the distal nerve stump degenerates (Waller, 1985). SCs detach from axons, dedifferentiate, proliferate, and acquire a repair phenotype (Yang et al., 2008) (Figure 1). Macrophages are recruited to the injury site to promote axonal debris clearance and to release cytokines and factors, thus contributing to creating a favorable microenvironment (Liu et al., 2019). Endothelial cells also participate in nerve regeneration via angiogenesis but also promoting SCs proliferation and migration (Meng et al., 2022). Interestingly, endothelial cells-derived exosomes boost and maintain SC repair phenotypes via miR199-5p and activation of PI3K/AKT/PTEN signaling pathway (Huang et al., 2023). While nerve regeneration is active as mentioned above, in the context of disease this process is constantly impaired. Differently from nerve trauma, which is usually caused by an acute injury in a specific site, peripheral neuropathies (PNs) typically start along the length of the nerve and can progress over time becoming chronic. PNs are usually caused by various genetic mutations, infections, autoimmune and metabolic manifestations, or chemotherapeutic side effects. Such genetic diseases can spare SCs but still cause abnormal myelin development (hypo or hypermyelination). Therefore, the pathomechanisms of PNs vary according to several factors: SCs involvement, and the nature or timing of myelin dysfunction.

### 1.1. Role of SCs in the peripheral nervous system

**Figure 2:**
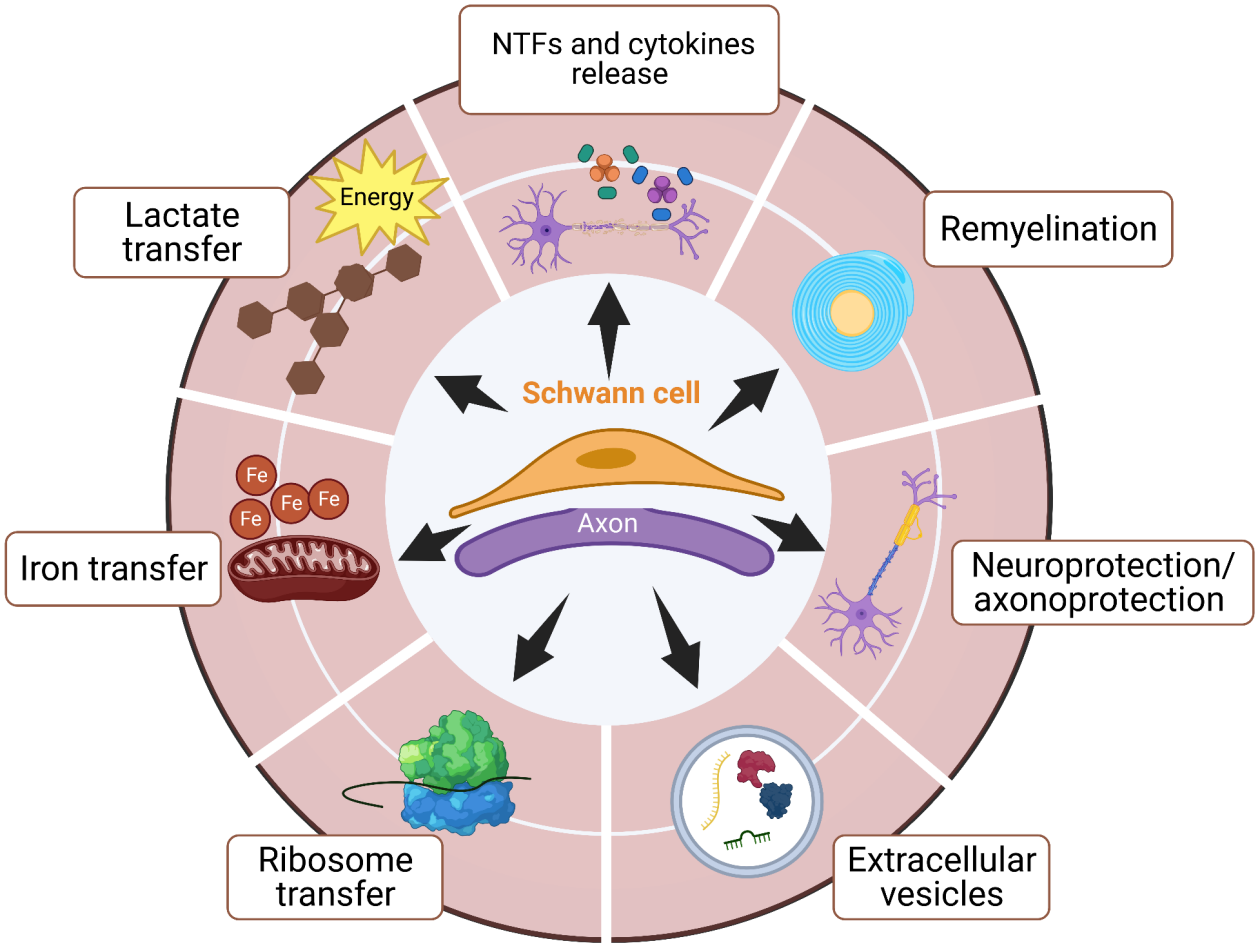
SCs support axons during development, homeostasis, and repair by several means. The plasticity of SCs allows these cells to have critical participation in supporting axons during development, maintenance, and nerve regeneration. Several functions have been identified in order for SCs to promote axonal regeneration after injury or disease onset The main known functions are neurotrophic factors (NTFs) and cytokines release, remyelination, neuroprotection/axonoprotection, secretion of extracellular vesicles for cell–cell or cell-axon communications, and also transportation of lactate and ribosome between SCs and axons.

SCs are the main supportive cells in the peripheral nervous system. Either with myelinating or non-myelinating phenotype, SCs support axons during development and are crucial for their function and maintenance. Following nerve damage, SCs, named repair SCs, dedifferentiate and activate a repair program to support nerve regeneration (Jessen and Mirsky, 2016). SCs act in the peripheral nervous system by several means, such as remyelination, direct neurotrophic factors release, neuroprotection/axonoprotection and transfer of extracellular vesicles, iron, lactate, and ribosomes (Figure 2). These mechanisms are crucial to understand interactions between SCs and neurons during homeostasis but also after trauma or disease onset.

#### 1.1.1. Remyelination

Myelination occurs during nervous system development and is maintained in health nerves. Efficient myelin debris clearance after injury or disease onset is crucial for regeneration to take place. Repair SCs are the local primary phagocytic cells, breaking down their own myelin in a process called myelinophagy, which is regulated by JNK/c-Jun pathway (Gomez-Sanchez et al., 2015). Repair SCs also trigger a local innate immune response by releasing several cytokines. In particular, this behavior is important to attract and activate neutrophils and macrophages, the latter taking over phagocytosis of axonal and myelin debris. Repair SCs guide axon regrowth to the target site by forming bands of Büngner, and once lined up with axons, start remyelination. In chronic myelin dysfunction, the number and repair capacity of SCs decreases over time (Sulaiman and Gordon, 2009). In addition, in some diseases, such as Charcot–Marie–Tooth (CMT) disease and chronic inflammatory demyelinating polyneuropathy, the constant cycle of demyelination and attempts of regeneration forms dysfunctional layers of myelin, and forms concentric layers of SC processes and collagen surrounding axons (Smith et al., 1980 Snider, 1994 Midroni and Bilbao, 1995).

#### 1.1.2. Trophic support

It’s well-studied that repair SCs supply trophic factors to axons since development for guiding the sprouting growth cones, and for maintenance and repair. Studies using neurotrophic factor knockout mice highlight the importance of these molecules since they show lethal or dysfunctional phenotypes. For example, brain-derived neurotrophic factor heterozygous mice develop an eating behavior disorder and abnormal locomotor activity (Kernie, 2000), while the knockout mice phenotype present loss of sensory and sympathetic neurons and die within the first month of life (Crowley et al., 1994). Among some trophic factors released by repair SCs to axons are nerve growth factor (Bandtlow et al., 1987; Meyer et al., 1992), brain-derived growth factor (Meyer et al., 1992), ciliary neurotrophic factor (Meyer et al., 1992), vascular endothelial growth factor (Höke et al., 2006), hepatocyte growth factor (Höke et al., 2006), neurotrophin-3 (Sahenk et al., 2008), pleiotrophin (Mi et al., 2007), and insulin-like growth factor (Syroid et al., 1999). Interestingly, non-myelinating and myelinating SCs display different neurotrophic factor type release because some factors play a more important role than others depending on if the nerve is mainly sensory or motor. For example, pleiotrophin is crucial for motor nerve regeneration while sensory nerves require brain-derived and nerve growth factors for regeneration (Höke et al., 2006). These molecules can be released to axons by two means: through extracellular vesicles or as soluble factors not loaded in vesicles.

#### 1.1.3. Neuroprotection/axonoprotection

The neuronal body in the spinal cord and in the dorsal root ganglion is also dependent on neurotrophic support from the glia for survival, maintenance, and repair. SC-derived neurotrophic factors are conveyed to the neuronal body by retrograde axonal transport (Davies, 1998) supporting neuronal survival. *In vitro* studies using SC-conditioned media on motor neurons show that repair SCs support neuronal survival by providing neurons with neurotrophic factors (Bosch et al., 1988; Wu and Zhu, 1996).

There is a lack of significant neuronal death in most peripheral neuropathies, with axons and myelin being mostly affected. On the other hand, following nerve injury, several morphological and molecular changes take place in the cell body in an attempt to recover but significant cell death by apoptosis or necrosis may occur (Liu and Wang, 2020). Not only affected neurons, but SCs distal to the injury change their gene expression profile. While SCs switch to a repair phenotype, neurons switch from signaling to a growth mode.

Therefore, regeneration signaling pathways take place in injured axons and neuroprotection signaling pathways take place in affected neuron bodies. In a study using *in vivo* and *in vitro* models, they showed that in response to axonal injury, the increase in nitric oxide levels by affected neurons triggered periaxonal SCs to release erythropoietin which, via its receptor binding on neurons, prevented axonal degeneration (Keswani et al., 2004). The authors concluded that this signaling pathway provided protection against injury to both the cell body and the axon. The potential of erythropoietin to protect the axon and neuron body from degeneration was also demonstrated *in vivo* studies of chemotherapy drug-induced peripheral neuropathy (Melli et al., 2006) and diabetic neuropathy (Bianchi et al., 2004).

#### 1.1.4. Extracellular vesicle release

Extracellular vesicles are lipid bound spheres secreted by cells into the extracellular space that act as a form of intercellular communication. Extracellular vesicles have different subtypes characterized based on subcellular origin, size, and composition (Verweij et al., 2021). The exosomes are a very small subtype of extracellular vesicles of endosomal origin that contain complex cargo including trophic factors, proteins, lipids, metabolites, and nucleic acids such as mRNA, miRNA, and DNA (Isola and Chen, 2017). SCs transfer exosomes to nerves during homeostasis as well as following trauma or disease. Exosome cargo varies according to the SCs phenotype and to the physiological or pathological microenvironment (Sohn et al., 2020).

It has been shown that exosomes from SCs have a neuroprotective effect on neurons by blocking the caspase-3 cell death pathway (Hyung et al., 2019). Exosomes also regulate inflammation after injury. Following rat spinal cord injury, SC-derived exosomes containing MFG-E8 as the major component suppressed M1 polarization and stimulated M2 polarization, which in turn inhibited neuronal apoptosis (Ren et al., 2023). The exosomes were phagocytosed by macrophages/microglia and released MFG-E8, causing an increase in the protein levels of SOCS3, which inhibited the phosphorylation of STAT3. The authors concluded that activation of SOCS3/STAT3 pathway participated in upregulating M2 polarization for alleviating inflammatory damage.

The important role of exosomes on axons could become more evident in diseases. For example, mutations in CMT1 genes encoding the small integral membrane protein of the lysosome/late endosome, which regulates the production of exosomes. Mouse primary embryonic fibroblasts, patient B cells, and mouse primary SC CMT1 cells show a decreased number of exosomes, interfering in intercellular communications, which may account for CMT1 molecular pathogenesis (Zhu et al., 2013).

#### 1.1.5. Ribosome transfer

SCs are also organelle suppliers for axons Court et al. (2008), using the Wallerian degeneration mouse model, demonstrated that SCs can control axonal protein synthesis by transferring polyribosomes to it through resulting vesicles. Interestingly, both injured and intact axons, even to a lesser extent, were supplied by SCs.

#### 1.1.6. Iron transfer

Neurons are cells with high metabolic demands. SCs play a role in axonal metabolic support by providing iron to axonal mitochondria. Mietto et al. (2021) showed that mouse SCs express the molecular machinery to release iron and that both axons and SCs express the iron importer transferrin receptor 1, which implies that they can uptake iron. In addition, lack of iron export from SCs in ferroxidase ceruloplasmin knockout mice reduced mitochondria iron in axons and impaired axonal regeneration following nerve injury. The authors showed that in response to axonal injury, the increase in nitric oxide levels by affected neurons triggered periaxonal SCs to release erythropoietin which, via erythropoietin receptor binding on neurons, prevented axonal degeneration (Keswani et al., 2004).

#### 1.1.7. Lactate transfer

Although not completely clear, there is evidence that SCs metabolically supply axons by providing them with lactate. Through aerobic glycolysis, SCs metabolize glucose into lactate and shuttle it to neurons. Pyruvate kinase 2, which catalyzes the dephosphorylation of phosphoenolpyruvate to pyruvate, is upregulated in myelinating SCs of mature mouse sciatic nerve, and its genetic deletion led to a deficit of lactate in the nerve. The mutant mice with the deletion of pyruvate kinase 2 in myelinating SCs developed peripheral neuropathy with impaired production of ATP, slow mitochondria transport, retraction of muscle axon terminals, and motor neuron stress (Deck et al., 2022). Therefore, the supply of lactate by SCs is crucial for long-term maintenance of axonal function and for supporting axons following injury or disease onset.

The glycolytic upregulation in SCs paired with enhanced axon-glia metabolic coupling in response to axon injury supports axon survival. Takenaka et al. (2023) showed that following axotomy, the glycolytic system in SCs and axons, and monocarboxylate transporters-induced monocarboxylate transport to axons were activated in the distal stump. They suggested that pyruvate is transported from SCs to axons through monocarboxylate transporters and metabolized promoting a shift towards the glycolytic system to produce ATP.

Interestingly, they also suggested that SCs worked as a lactate buffer, receiving the excess produced lactate in axons, and releasing it through extracellular space via monocarboxylate transporters. The critical role of the monocarboxylate transporters in axonal bioenergetics was also demonstrated in the monocarboxylate transporter 1 null mouse crush injury model (Morrison et al., 2015). In this study, the authors demonstrated that the lactate transport was compromised in these mice, delaying the regeneration of both sensory and motor axons, probably due to dysfunctional lactate transport in SCs. Babetto et al. (2020) showed that the glycolytic shift in SCs-protected injured axons was driven by mTORC1 signaling and downstream transcription factors Hif1α and c-Myc which, in turn, promoted glycolytic gene expression. Interestingly, transection of all axons, but not axonopathy induced by oral administration of acrylamide, led to significant induction of mTORC1 activity. By genetically inducing sustained mTORC1 hyperactivity in SCs in this mouse model of axonopathy, the authors demonstrated the importance of this signaling pathway for conferring axon protection following axonopathy onset.

The complex roles of lactate metabolism in axons are not completely clear. Upstream of the mTORC1 signaling pathway is its activator Rheb, which regulates mitochondria metabolism through pyruvate dehydrogenase (Yang et al., 2021). Jia et al. (2021), using a SC-specific Rheb knockout mouse, showed that Rheb is critical for the metabolic coupling between SCs and axons through lactate but Rheb deletion increased lactate production and release. They concluded that lactate metabolism of neurons is beneficial to injured axons in the short term, but its persistence enhances reactive oxygen species production causing mitochondria damage and leading to impairment in axon stability. Therefore, the role of SCs in axonal energy homeostasis might be complex, not only for providing axons with lactate but also ensuring that this fuel will be provided in the proper amount and time and buffering it when necessary.

Several molecular targets, drugs, biomaterials, and cellular transplantation procedures have been developed, showing the potential to improve nerve regeneration following injuries and neuropathies. However, there are still challenges to moving potential therapies toward clinical translation studies. Although various studies have shown progression in understanding the role of SCs-axon interaction and the pro-regenerative process by using novel compounds, there is a need for efficient *in vitro* models supporting the transition from preclinical studies to translational studies. Therefore, in the next section, we discuss *in vitro* models to generate SCs by direct reprogramming or from induced pluripotent stem cells (iPSCs). Furthermore, we focus on studies using protocols able to mimic the SCs-axon interaction *in vitro,* and preclinical studies *in vitro* in the context of neuropathies.

## 2. Preclinical studies and *in vitro* protocols to investigate SCs-neuron interaction

**Figure 3:**
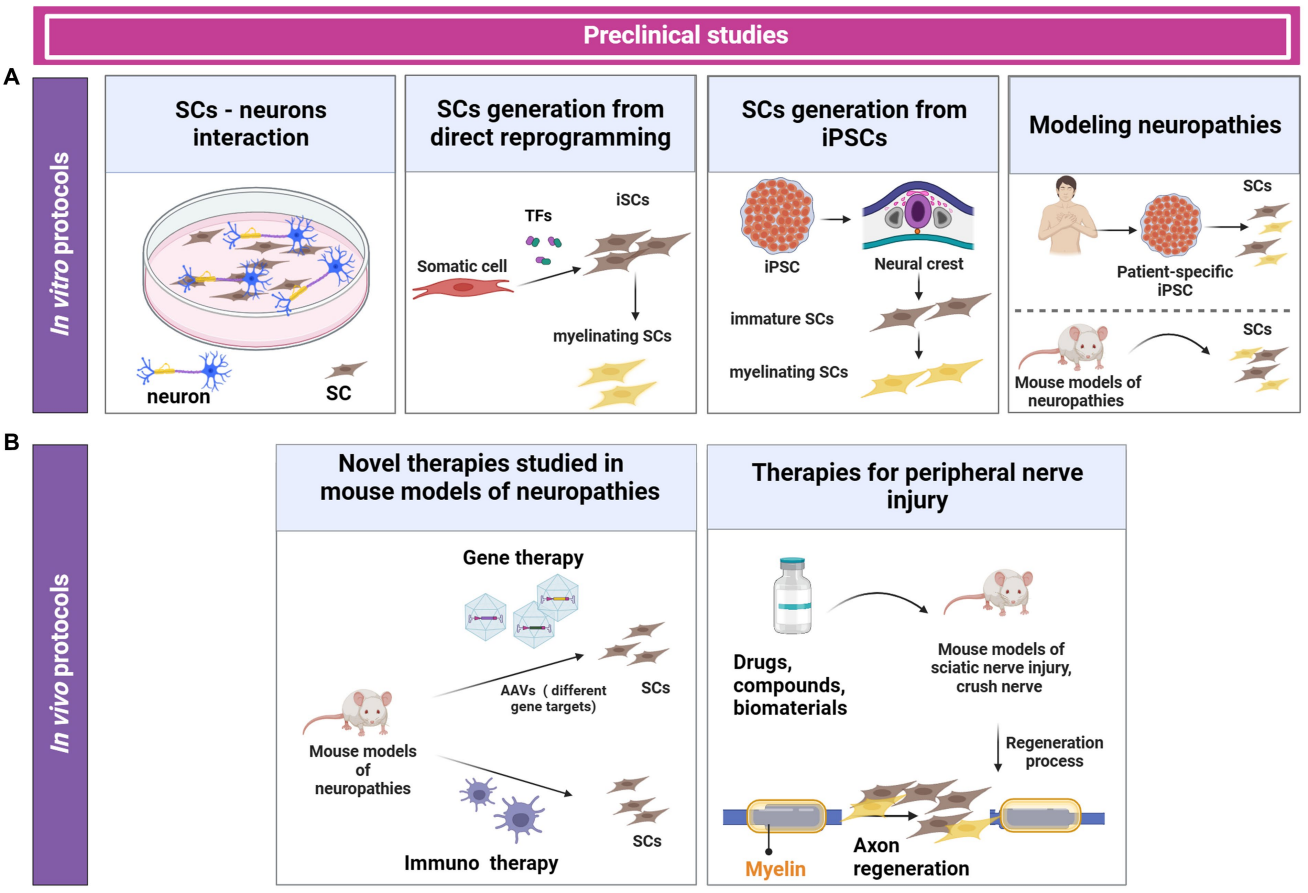
*In vitro* and *in vivo* protocols for generating SCs and investigating SC-neuron interaction. *In vitro* protocols use primary SCs, SC lines, SCs generated from direct reprogramming, or SCs derived from iPSCs. These *in vitro* protocols are used to investigate candidate therapeutic drugs and to study pathomechanisms of neuropathies **(A)**. *In vivo* preclinical studies involve the use of animal models of neuropathies and peripheral nerve injuries. Currently, different therapies such as gene therapy and immunotherapies have been explored in preclinical trials **(B)**.

During the last few decades, several *in vitro* protocols have been designed to use primary SCs or SC lines or to generate SCs either through direct reprogramming or from iPSCs (Zhu et al., 2013). These models allow the study of SC-axon interaction because they mimic the nerve microenvironment *in vitro*. *In vitro* models can be divided into two systems according to the number of cell layers: two-dimensional (2D) culture, and the more complex three-dimensional (3D) co-culture. These *in vitro* models are useful tools to model aspects of peripheral nerve injury and neuropathies, and it allows for high-content drug screening (Figure 3A) (Rayner et al., 2018).

### 2.1. Two-dimensional *in vitro* models

#### 2.1.1. *In vitro* protocols of SCs isolated from tissues or cell lines

Several protocols have been established to isolate SCs from healthy or neoplastic tissues from neonatal and adult rats, mice, and large animal’s nerves. In addition, primary human SCs have been isolated from the cauda equina of health donors or patients, and have been used to study nerve regeneration, drug screening, tissue engineering, disease modeling, among others.

Due to the fact primary cells have limited proliferation potential and develop senescence (Monje, 2020), there is a challenge in obtaining sufficient cell numbers *in vitro*. To circumvent this limitation, SCs can be immortalized or isolated from neoplastic tissues (Li et al., 2022).

Immortalized cell lines derived from peripheral glia have been used in several studies. The RT4-D6P2T cell line is a SC derived from rat schwannoma and presents similar characteristics to primary SCs compared with other cell lines. This is due to RT4-D6P2T expressing key genes associated with myelination (Hai et al., 2002). However, they do not express ErB type I/II as primary SCs do (Fricker and Bennett, 2011). Therefore, the lack of these receptors can impact the SC-axon interaction. Other SC lines used in cell cultures are JS-1 (Kimura et al., 1990), RSC96 (Badache and De Vries, 1998), R3 (Li et al., 1995; Hai et al., 2002), S16Y (Toda et al., 2002) and SCL4.1/F7 (Hai et al., 2002). These SCs are either obtained from normal tissues, tumors of nervous tissues, or from genetic manipulation of glial precursors.

Although immortalized or induced SCs can be expanded *in vitro*, SCs isolated from humans or mice show low myelination efficiency in most of *in vitro* protocols (Monje et al., 2018; Balakrishnan et al., 2021). While SCs isolated from rats can get to a mature stage to myelinate in co-culture with neurons or dorsal root ganglion (DRG), SCs isolated from mice and humans show inefficient myelination potential (Tao, 2013) (Figure 3A). Therefore, there is a need to improve the generation of human SCs with high myelination capacity.

#### 2.1.2. SCs generated by direct reprogramming

Other emergent alternatives such as direct reprogramming can offer a sufficient number of functional SCs able to myelinate neurites *in vitro* (Sowa et al., 2017). Nerve regeneration studies are mostly based on the interaction between neurons and SCs. SCs are the main supportive cells in this process. Because SCs derived from primary cells are extremely limited, an alternative is to generate induced SCs (iSCs) from somatic cells by direct reprogramming, without passing through the pluripotent state (Figure 3A).

Several *in vitro* differentiation protocols have been established using small molecules (Thoma et al., 2014; Kitada et al., 2019), or by overexpression of the transcription factors in combination or not with extracellular matrix components. These protocols describe cell isolation, enrichment, differentiation, and characterization, and functional experiments such as the capacity of SCs to myelinate axons and their outcomes on regeneration (Kim et al., 2020; Hörner et al., 2022) (Table 1).

**Table 1:** Transcription factors of direct reprogramming from somatic stem cells to generate iSCs precursors and mature iSCs.

Somatic cells	Transcription factors	Growth factors	System used	Positive cells converted	Functional experiments	Reference
Mouse, rat, human fibroblast	Sox10, Krox20	Forskolin, NGF1	Doxycycline-inducible vectors lentivirus	12-5% S100/O4 positive cells	iSCs co-cultured with mouse DRG	Mazzara et al. (2017)
Human skin fibroblast	Sox10, Krox20, Oct6, Klf4, Oct4, c-Myc	Forskolin, HRG-b1, NGF1	Retrovirus vectors	43% of SCs	iSCs co-cultured with mouse DRG or NG108-15 cells. iSCs successfully transplanted *in vivo*. Sciatic nerve innervation	Sowa et al. (2017)
Human skin fibroblast	Oct3/4, Klf4, Sox2, L-Myc, Lin28, p53 shRNA	NGF1, PDGF-BB	Episomal vectors	95% Sox10 positive population	iSCs co-cultured with mouse DRG or human iPSC-derived sensory neurons. Reinnervation *in vivo*	Hörner et al. (2022)

In recent years, studies have directly generated rabbit, mouse, and human iSCs from fibroblasts or mesenchymal cells *in vitro* by introducing transcription factors associated with SCs differentiation, such as Sox10 (sex-determining region Y-box 10), Krox20/Egr2 (Wang et al., 2011; Sowa et al., 2017), Oct6 or Sox10 in combination with Krox20/Egr2 (Mazzara et al., 2017). Other transcription factors used for SC differentiation are Oct3/4, Klf4, Sox2, L-Myc, Lin28, and p53 shRNA, which result in high efficiency of Sox 10 population cells. Interestingly, directly iSCs improved sciatic nerve regeneration *in vivo* and provided reinnervation of gastrocnemius muscle after transplantation in mouse (Sowa et al., 2017; Kim et al., 2020; Hörner et al., 2022).

Recent advances in the direct reprogramming of murine primary cells to SCs have provided a supportive platform for screening SCs-specifying TFs, allowing the discovery of new candidates. However, there is not enough evidence demonstrating that iSCs are able to successfully myelinate axons, among other critical functions. Several of these protocols are time-consuming and generate differentiated SCs-like cells with low myelination efficiency limiting the development of cell-based therapies and *in vitro* disease-modeling studies.

In the last few decades, several key transcription factors were found to have an important role in the transition of immature SCs to a mature state able of neuronal interaction (Table 2). Besides the Sox10 and Krox20, other transcription factors and proteins have been associated with myelination such as Pmp22, p75, such as Pmp22, p75, P0/Mpz and S100B (Topilko et al., 1994; Bondurand, 2001; LeBlanc et al., 2007; Fricker and Bennett, 2011; Donato et al., 2013; Cervellini et al., 2018; Fogarty et al., 2020; Gonçalves et al., 2020).

**Table 2:** Proteins involved in immature and mature SCs stages and neuronal interaction.

Protein	Main function	Type of cell	Reference
S100B	Proliferation, differentiation, apoptosis, calcium homeostasis, energy metabolism, inflammation response, and migration through interactions with several target proteins including enzymes, cytoskeletal subunits, receptors, transcription factors	Immature and mature SCs	Donato et al. (2013)
GALC	Involved with macrophage interaction to perform myelin debris degradation	SCs interaction with macrophages	Weinstock et al. (2020)
GFAP	Proliferation	Development, non-myelinating SCs, immature SCs	Chen et al. (2007)
KROX20	Activated by Oct6 and Brn2, acting in the promyelinating stage blocks proliferation of SCs	Myelinating SCs	Topilko et al. (1994)
p75 (NGFR)	Neuroprotein receptor	Myelinating SCs	Gonçalves et al. (2020)
PLP	Conform the major structural myelin. It is required for the long-term preservation of myelinated axons	Myelinating SCs	Patzig et al. (2016)
PMP22	Preferentially expressed in PNS	Myelinating SCs	Mittendorf et al. (2017)
ERBB2/ERBB3	Neurotic network to myelination, promyelin TF, cell migration receptor of nrg1	Myelinating SCs	Lyons et al. (2005)
P0/MPZ	Promyelin TF, associated with Sox10 and Egr2	Myelinating SCs	LeBlanc et al. (2007)
Cnx32	Directly regulated by Sox10	Myelinating SCs	Bondurand (2001)
Oct-6/Pou3f1	Promyelin TF, when inhibited there is a transient delay in myelination	SCPs precursors	Donato et al. (2013) Topilko et al. (1994)
Brn2/Pou3f2	Promyelin TF, compensates lack of Oct-6	SCPs precursors	Donato et al. (2013)
NFkB	Promyelin TF	SCs	Morton et al. (2013)
Sox10	Development/promyelin TF, new targets: ARPC1A, CHN2, DDR1, GAS7 (genes associated with PNS)	SCs	Fogarty et al. (2020)
Nab 1 and 2	Receptors of Egr2, repress Erg2	SCs	Srinivasan et al. (2007)
Nrg1	SC growth and differentiation	Neuron interaction with SCs	Fricker and Bennett (2011)
Prx	Promyelin TF encodes 2 PDZ-domain proteins, required for maintenance of peripheral nerve myelination	SCs	Wang et al. (2018)
Cnp, Mal, Mapk3	Differentiation and myelination	SCs	Cervellini et al. (2018)
Mbp	Promyelin TF	SCs	García-Suárez et al. (2009)

S100B, S100 calcium Binding protein b; GALC, galactosylceramidase; GFAP, glial fibrillary acidic protein; NGFR, nerve growth factor receptor; PMP22, peripheral myelin protein 22; PLP, proteolipid protein 1; Erbb3, Erb-B3 receptor tyrosine kinase 3; P0/Mpz, myelin Protein 0; Cnx32, connexin32; Oct-6/Pou3f1, Oct-6/POU domain, class 3, transcription factor 1; NFkB, nuclear factor – kB; Sox10, SRY-box transcription factor 10; Nab1 and 2, NGFI-a-binding protein 1 and 2; Nrg1, neuregulin 1; Prx, periaxin; Cnp, C-type natriuretic peptide; MaL, myelin and lymphocyte protein; Mapk3, mitogen-activated protein kinase 3;MBP, myelin basic protein.

After SC differentiation, iSCs can be used for *in vitro* myelination assays performed in co-cultures with DRG, for example. In addition, iSCs generated by direct reprogramming can graft in rabbit nerve, showing remyelination and regeneration *in vivo* (Wang et al., 2011; Clark et al., 2017; Kim et al., 2020).

#### 2.1.3. SCs derived from iPSC

SCs can be generated from several stem cell sources, including adult tissue, iPSC, and ESC. The main advantages of using iPSCs are the ethical regulation to use this type of cells compared with embryonic stem cells, and the unlimited self-renew potential. Currently, human iPSCs are used as a potential cell therapy approach. Although SCs derived from iPSC show low capacity of myelination *in vitro*, there are protocols showing that after transplantation these SCs are functional *in vivo*. Thus, the nerve environment has an important role in the transition from immatures SCs to mature SCs. Also, they can differentiate into neural crest (NC) cells and provide motor and sensory neurons, and SCs (Kim et al., 2017).

The first step of this protocol, named the “early stage,” includes neuroectodermal differentiation and NC formation. The second step, named “late stage,” focuses on SCs generation, which includes immature and mature stages. NC induction is regulated by specific signaling pathways associated with early neural development such as Wnt, FGF, BMP, and Notch pathways. Specific transcription factors are expressed in NC inductions dictating cell migration and differentiation. Although NC induction involves multistep gene regulation, the activation of canonical Wnt pathway drives this differentiation (Mica et al., 2013). In Xenopus, the Wnt receptor is responsible to regulate the expression of *Sox10*, *Sox9*, *Snail*, *twist* and *Foxd3*, which are key genes for the successful of NC generation (Ji et al., 2019). Recently, studies suggested that YAP signaling pathways may play an important role in migration together with the signaling pathways mentioned above (Hörner et al., 2022).

In addition, during embryonic development, Bmp, Yap and Retinoic acid interact with Wnt1 participating in the trunk NC cell emigration. Yap signaling is associated with Bmp and Wnt signaling regulation by inducing G1/S transition in the cellular cycle, proliferation, survival, and is involved in delamination of pre-migratory NC progenitors (Kumar et al., 2019). Furthermore, Fgf signaling pathway can regulate *Noggin* expression in the caudal neural tube and Bmp blocks the NC cell delamination. Thus, Fgf is a timing regulator of the delamination. In this context, SCs precursor initially can differentiate from NC (early embryonic nerve) and then differentiate as an immature SCs stage (later embryonic nerve). SC precursors, and motor and sensory neurons can differentiate from the trunk neural crest. The *in vitro* protocols can recapitulate stages of *in vivo* embryonic development, therefore, the Wnt pathway regulation is critical to the NC induction. NC can be differentiated from iPSC in two-dimensional culture or from embryoid bodies. The embryoid bodies will be induced to generate neurospheres or organoids. However, there are limits to using these protocols due to the generation of a mixed neural population.

Recently, other protocols in which SCs have been differentiated from iPSCs using transcription factors or small molecules such as inhibitors of TGF-β, GSK-3β, and NRG1 or inhibitors of multiple kinases were established (Thoma et al., 2014; Kim et al., 2017). To induce neuroectodermal differentiation, several protocols utilize the inhibition of GSK-3β, which activates the Wnt signaling pathway. The most used inhibitors are CHIR99021 and SB431542. Other protocols use BMP pathway inhibitors such as LDN193189 to induce neural crest formation. In addition, epidermal and basic fibroblast growth factors are added in some protocols to more efficiently generate the neural crest (Ziegler et al., 2011; Kim et al., 2017; Muller et al., 2018; Carrió et al., 2019; Mukherjee-Clavin et al., 2019). Several protocols use these inhibitors, which are used in different concentrations, and added or completely removed at different time points.

For the late stage, to generate neural crest migration, SCs precursors, immature SCs, and myelinating SCs there are protocols using small chemicals such as neuregulin 1, forskolin, fetal bovine serum, basic fibroblast growth factor, and retinoic acid among others. Despite the protocols showing SCs able to myelinate *in vitro* and *in vivo* there are challenges such as SCs isolation and enrichment, and still low capacity to become functional myelinating SCs. The use of basic fibroblast growth factor in the late stage of induction plays an important role in increasing the proliferation and survival of immature SCs. Therefore, most of protocols include this factor (Ziegler et al., 2011; Kim et al., 2017; Muller et al., 2018; Carrió et al., 2019; Mukherjee-Clavin et al., 2019).

The generated SCs-like can be characterized by two steps. First, evaluation of the SC phenotype in terms of protein expression and gene expression profile between day 50 to 90 in culture. Then, co-culturing SCs with motor neuron neurospheres from iPSC to evaluate the capacity of the generated SCs to myelinate neurons (Liu et al., 2012; Huang et al., 2016; Shi et al., 2018).

Another important factor to consider in protocols for generating functional SCs is the extracellular matrix. While some protocols use Geltrex or Matrigel, others use laminin, collagen, among others (Ziegler et al., 2011; Kim et al., 2017; Muller et al., 2018; Carrió et al., 2019; Mukherjee-Clavin et al., 2019).

Studies modeling neuropathies *in vitro* have been done using patient-specific iPSCs. These studies have used the protocols mentioned above and were able to recapitulate characteristics of the disease *in vitro*. Due to CMT having different types of mutations, the iPSCs models allow the evaluation of the biological function of the different mutations *in vitro*. Modeling neuropathy *in vitro* also allows the evaluation of motor and sensory neuron functions, mitochondrial dysfunction, myelin production, among other biological processes compromised in the specific disease studied (Figure 3A) (Saporta et al., 2015; Clark et al., 2017; Saporta and Shy, 2017; Perez-Siles et al., 2020; Van Lent et al., 2021; Ababneh et al., 2022; Hörner et al., 2022). For example, a recent study recapitulated diabetic peripheral neuropathy *in vitro* by using iPSCs. iSCs were derived from iPSCs and the authors performed a high-throughput drug screening. The study identified that bupropion could prevent sensory neuron dysfunction in a mouse model of diabetic peripheral neuropathy (Majd et al., 2023).

Despite *in vitro* protocols being useful tools to study processes such as SC proliferation, migration, and myelination, axonal projection, and production of key neurotrophic factors, it is still necessary to optimize protocols for obtaining higher numbers of functional myelinating SCs *in vitro*.

#### 2.1.4. *In vitro* protocols to promote myelination

During the last few decades, several *in vitro* protocols were established to improve the capacity of SCs to myelinate. Most common protocols use primary SCs in co-culture with neurons.

Due to the interaction between SCs and neurons playing a crucial role in PNS regeneration, co-culture systems are necessary. Immortalized neuronal cell lines such as PC12, a neural cell line derived from pheochromocytoma of rat adrenal medullary, have been used for this purpose. This cell line shows a pro-regenerative potential when several compounds are tested in neurite outgrowth assays (Greene and Tischler, 1976; Haastert-Talini et al., 2014). 50B11 is a sensory neuron cell line isolated from rat embryonic DRG (Chen et al., 2007). This cell line is also used for neurite outgrowth assays (Li et al., 2022).

The NSC34 cell line is a motor neuron generated from mouse neuroblastoma cells and motor neuron-enriched spinal cord cells combined (Cashman et al., 1992). The main advantage is that unlike primary neurons, these cells can be expanded for a long time *in vitro*. Also, this cell line can respond to neurotrophic factors and hormones. NG108-15 is a neuroblastoma cell line derived from the brain of a rat often used in several studies in co-culture with SCs cell lines (Ching et al., 2018).

SCs can be co-cultured with DRG for an average of 21 days. During this period, myelination capacity of SCs and axonal regeneration can be evaluated. Even though *in vitro* it is not possible to identify the bands of Büngner observed *in vivo*, it is still possible to evaluate myelination by using myelin protein markers. Novel compounds have been found to improve the SCs function and to support successful transplantation *in vivo*, being attractive therapeutic targets (Juan et al., 2020; Balakrishnan et al., 2021).

A conventional protocol published used a co-culture of motor neurons and SCs seeded on matrigel. The results showed that neurite growth increased after 3 weeks. SCs were able to myelinate the axons, and myelin production was identified by ultrastructural analysis. In addition, *in vitro* administration of antioxidant Co-Q10 (1 uM) for 24 h in SCs cultures showed that Co-Q10-treated mSCs increased the myelination capacity, which was quantified by MBP expression (Hyung et al., 2015).

Another alternative protocol is to inhibit the epigenetic regulator HDAC3. HDAC 3 inhibitor is able to increase myelin production and improve axon regeneration. Evidence showed that mouse SCs deficient in HDAC3 presented improved peripheral myelin repair. Studies showed that HDAC3 is a negative regulator of NRG1 and PI3K-AKT-mTOR signaling pathways. The authors suggested that HDAC3 inhibitors can be a therapeutic agent for peripheral neuropathies and nerve trauma (He et al., 2018).

Recently, new drugs able to inhibit MEK-ERK pathway have been showing an attractive option to improve the SCs differentiation *in vitro*. A study demonstrated that Dabrafenib (10 uM), a Raf inhibitor, was able to inhibit the ErK pathway and increase ErbB2 autophosphorylation and Akt phosphorylation. These results together suggest that ErbB2-PI3K-Akt has an important role to promote SCs differentiation (Park et al., 2021).

Another study evaluated the potential of BACE1, an enzyme able to generate β-amyloid peptides, to promote the proliferation and migration of SCs. BACE1-null mice presented hypomyelination. The authors showed that BACE1 can regulate the JAgged-1 and Delta 1 membrane ligands of Notch. The study demonstrated that BACE1-null mice are not able to uptake lactate as an alternative energy source. This mouse model also showed an increased SCs proliferation, with shortened internodes through the axon, and reduced myelin production associated with the dysregulation of nrg1-Erbb signaling (Hu et al., 2017).

In several neuropathies, there is a failure to produce normal myelin levels. More than 100 mutations in specific genes in SCs are responsible for this phenotype in patients with CMT. CMT is the most common neuropathy affecting myelin production. For the last decade, different potential therapeutic agents have been suggested based on molecular mechanisms associated with myelin production. Nrg1 type III plays an important role in myelination stages by interacting with receptor Erbb2 and regulating the expression of Krox20/Erg2, which are key transcription factors for regulating the expression of genes associated with myelination and lipid production, such as Mpz, and Mbp. Thus, Nrg1 is a potential target to treat neuropathies, in particular CMT. A study demonstrated that the overexpression of Nrg1 can increase the myelin thickness, without influencing the demyelination stage. In addition, the authors observed that the overexpression of Nrg1 type III can activate genes independent of the Krox20 pathway. This study suggested the use of TACE/ADAM17 (Nrg1 III inhibitor tumor necrosis factor—alpha-converting enzyme) can modulate Nrg1 Type III activity to regulate myelin production (Scapin et al., 2019).

### 2.2. Three-dimensional *in vitro* models

The 3D *in vitro* models can recapitulate the cellular organization and nerve environment observed in the peripheral nerve, allowing a better comprehension of repair and regeneration processes after an injury. Several 3D culture models include the use of biomaterials such as hydrogels and SCs delivery, engineered tissues, self-assembled aggregate cultures, and scaffold-based cell cultures. In SCs 3D cultures, the extracellular matrix such as collagen and laminin play an important role in the axonal growth assay (Rayner et al., 2018).

Recently, a comparison study evaluated the effect of ibuprofen on neurite regeneration *in vitro* using 2D or 3D culture models. The 2D model used SC line SCL4.1/F7 or neuronal cells (PC12, NG108-15, and DGR), while 3D models included engineered neural tissues, collagen gel, and SCs cell lines in co-culture with neuronal cells. The authors also focused on generating a 3D model *in vitro* to study drug screening, for example evaluating the effect of ibuprofen on neurite regeneration *in vitro*. The results showed that both culture systems (2D and 3D) were able to identify a significant difference in the total number of neurite formation. However, the 3D culture system showed results more consistent compared with *in vivo* models and less variability (Rayner et al., 2018).

The main advantage of 2D models is their simplicity and the low cost compared with 3D models or *in vivo* studies. However, 2D models fail in recapitulating the nerve environment. On the other hand, 3D models offer representative results due to their capacity to mimic cell-to-cell interactions observed *in vivo*.

## 3. Preclinical studies modeling peripheral neuropathies or peripheral nerve injury *in vitro* and *in vivo*

SCs have a crucial role in peripheral nerve regeneration in response to injury or neurophaties. The plasticity of SC precursors allows the transition from their early state to a mature state to generate myelinating SCs, which protect and insulate nerve fibers in the PNS (Jessen and Mirsky, 2019). During nerve regeneration, mature SCs can return to an immature state, generating SC precursors, followed by an active repair state. In this process, SCs can dedifferentiate to produce myelin. The interaction between neurites and SCs promotes axonal regeneration, in part because neurotrophic factors and cytokines are released from the cells in the nerve environment. *In vitro* models are extremely useful tools to mimic direct or indirect cellular (conditioned media) communication (Figure 3).

During the last decades, many molecular targets, neurotrophic factors, biomaterials, and drugs have been evaluated as potential therapeutic agents to improve nerve regeneration and nerve function, however, there are challenges to translating these promising therapies to clinical studies. Despite progress in understanding regenerative processes, there is a need for adequate protocols to evaluate nerve myelination due to their restricted number and lack of protocols, as well as, to evaluate the effect of therapeutic agents in a pre-clinical study.

Alternatively, the derivation of SC-like cells from multiple stem cell sources such as mesenchymal cells as well as humanized mice provide promising human models to study neuropathies. Several *in vivo* studies have shown nerve regeneration improvement by autologous cell engraftment using stem cells derived from tissues or iPSCs, combined or not with engineered materials. Furthermore, exosome delivery and key neurotrophic factors delivery have been studied in nerve regeneration. *In vivo* studies consider complex physiological responses for studying nerve regeneration providing results with potential translational research. Although in recent years these studies have shown progression, there is a lack of adequate, cost-effective, and reproducible protocols to perform *in vitro* and *in vivo* studies.

### 3.1. *In vitro models* to study peripheral neuropathies

*In vitro* studies allow investigating different therapeutic approaches, from drug and small molecules to transgene delivery in a less complex but more direct way. Although there are several therapeutic agents with potential to promote nerve regeneration or neuroprotection, most of them result in partial nerve recovery. Recently, several studies have suggested the use of a combinatorial strategy to target both neurons and SCs in order to improve the functional recovery by using small molecules or transcription factors (Balakrishnan et al., 2021).

### 3.2. Modeling peripheral neuropathies *in vivo*

Peripheral neuropathies models are an attractive tool to evaluate rare diseases such as Charcot–Marie–Tooth disease. Guillain–Barré syndrome, schwannomatosis disorders among others. Due to some patients carrying mutations in different genes, such as *Cx23* and p*mp22* in CMT patients, resulting in lack of function and dysfunctional SCs, it is possible to recapitulate the disease *in vitro* by iPSC differentiation. Gene therapy is an option to restore the function of these genes. However, it is necessary to consider the physiological response. Therefore, the use of mouse models for studying specific diseases allows a better understanding of safety and toxicity, as well as the identification of the minimum dose able to give a therapeutic effect *in vivo* (Table 3). Since the SCs can engraft *in vivo*, iPSCs-derived cell replacement could be an attractive alternative tool to transplantation (Figure 3B).

**Table 3:** Pre-clinical trials *in vivo* in gene therapy, small molecules treatment and immunotherapy.

Animal model	Mutation/type of cells	Drug/gene delivery	Type of administration	Major findings	Reference
Charcot–Marie–Tooth disease 1 A (CMT1A)	Duplication of the PMP22/SCs	AAV2/9.shRNA Pmp22	Intra-nerve injections	A bilateral treatment restores PMP22 expression. Increased myelination and prevention of motor and sensory neuron impairments over 1 year	Gautier et al. (2021)
Charcot–Marie–Tooth disease (CMT1X) -Gjb1/Cx32 null (Cx32 KO)	Mutations in the GJB1 gene encoding a protein connexin32 (Cx32)	AAV9.Mpz.GJB1	Lumbar intrathecal injection	Improved motor functions and increased SN conduction velocities along with increased myelination and reduced inflammation in PNS	Kagiava et al. (2021)
Charcot–Marie–Tooth disease (CMT1X)-Gjb1/Cx32 null (Cx32 KO)	Mutations in the GJB1 gene encoding a protein connexin32 (Cx32)	scAAV1.tMCK.NT-3	Intramuscular delivery	Neuroprevention improved electrophysiological and histopathological phenotype of Cx32 KO mouse model	Ozes et al. (2022)
Charcot–Marie–Tooth disease type 2A (CMT2A)	Mutations in Mfn2	Small-molecule, Ser378 mimic MFN2 Ser378		A mitofusin agonist normalized axonal mitochondrial trafficking within sciatic nerves of MFN2 Thr105 → Met105 mice	Rocha et al. (2018)
Diabetic peripheral neuropathy db/db mice and human SCs (HSCs)		Cinacalcet	Standard chow diet	Cinacalcet decreased apoptosis process and increased autophagy activity after 12 weeks	Chung et al. (2018)
Diabetic neuropathy mice STZ, db/db		Inhibition of Kv2.1 by SP6616 AAV9-Kv2.1-RNAi		The results revealed the potential of SP6616 in preventing the occurrence of diabetic foot	Zhu et al. (2020)
Guillain–Barré syndrome		Anti-GM1 antibody		Anti-GM1 antibody expressed to exclusively target GM1 in either SC or axonal membranes	Goodfellow and Willison (2016)
Guillain–Barré syndrome		Inflammatory cells		Perisynaptic SCs have an important role to clear axonal debris	Cunningham et al. (2020)

#### 3.2.1. CMT diseases

CMT is the most common hereditary neuropathy. CMT has been mainly classified as type 1 or type 2 according to nerve conduction velocities and cell type affected. CMT1 has a demyelinating phenotype due to SCs being mostly affected and, as a result, nerve conduction velocities are slow. On the other hand, CMT2 has an axonal phenotype being characterized by axonal degeneration with nerve conduction velocities typically being almost unaffected. Intermediate CMT phenotypes with overlapping features of both types also exist (Juneja et al., 2019). Therefore, CMT is a heterogeneous disease with several different genetic mutations (Lupski et al., 2010). Genetic mutations that cause CMT can be related to neuron-type specific genes or SC-type specific genes. In CMT demyelinating phenotypes, genes that encode for proteins related to SC function, such as myelin components (PMP2, PMP22, and MPZ), transcription factors (EGR2/Krox20), proteins that play a role in degradation pathways (LITAF/SIMPLE), extracellular matrix (FBLN5), or adhesion molecules (GJB1/CX32 and PRX) are affected (Street et al., 2003; Punetha et al., 2018). Mutations in one of these genes can cause axonal damage, most likely secondary to the alterations affecting myelinating SCs.

Despite the challenges, loss and gain of function studies have been modeling demyelinating CMT subtypes *in vivo*. Zebrafish, mice, and rats have been mimicking many of the pathological hallmarks of CMT disease. Won et al. (2020) performed a loss of function study by knocking down fbln5 in the zebrafish model. This mutation decreased SC proliferation and resulted in myelination defects. The authors suggested that fbln5 present in the extracellular matrix or secreted from mesenchymal stem cells binds to integrin activating RAC1, which leads to actin remodeling to modulate SC myelination.

The PMP22 is one of the most affected genes in CMT mutations, in particular, by its duplication. PMP22 is a transmembrane glycoprotein component of peripheral nervous system myelin that is crucial for myelin development, maintenance, and function. Transgenic rat and mouse models have shown how myelination is affected morphologically with peripheral hypomyelination and SC hypertrophy, and functionally with reduced nerve conduction velocities and gait abnormalities (Sereda et al., 1996; Juneja et al., 2019).

The diameter of axons might also determine whether they will be myelinated as well as the myelin thickness. The autosomal dominant trembler mutation mouse model carries a point mutation in the PMP22 gene and is characterized by severe hypomyelination and continued SC proliferation (Suter et al., 1992). In these animals, cytoskeletal abnormalities including neurofilament hypophosphorylation are present, features that are also found in CMT1 disease. Watson et al. (1994) showed axonal cytoskeleton abnormalities, such as hypophosphorylation of neurofilament proteins, in nerve biopsies from patients with CMT1.

The authors concluded that SC-axon interaction may prevent full differentiation of the axonal cytoskeleton of myelinated nerve fibers, which resembled that of immature nerve fibers. Interestingly, SC modulation of neurofilament phosphorylation, axonal caliber, and slow axonal transport under demyelination was demonstrated in another study in which authors grafted autosomal dominant trembler mutation mouse nerve into the normal sciatic nerve (de Waegh et al., 1992).

To date, there is no effective treatment available for CMT. The therapeutic approaches investigated in *in vivo* studies have focused on gene therapy or small molecules (Stavrou et al., 2021) (Table 1). For example, adeno-associated viral (AAV) vectors 2 and 9 for GJB1/Cx32 delivery (Kagiava et al., 2021) or PMP22 silencing (Gautier et al., 2021) targeting SCs have shown great promise for treating CMT. Several small molecules have also been investigated for treating CMT, including ascorbic acid (Passage et al., 2004), which is a key factor for myelination. Despite great advances in the field, some therapeutic approaches translated to clinical trials have failed to show the expected efficacy. Since CMT is a complex hereditary demyelinating neuropathy with different aspects affecting axons and SCs, combining therapeutic approaches with different mechanisms of action might improve the outcomes observed in clinical trials.

#### 3.2.2. Diabetic neuropathy

Diabetic neuropathy is characterized by damage to nerve components, including axons, SCs, and blood vessels, caused by diabetes. Sensory nerve fibers are primarily and mostly affected followed by motor nerve fibers, leading to sensory deficits and muscle weakness, respectively. Autonomic nerve fibers can also be affected causing multiple organ dysfunction. Along with the direct effects of diabetes on neurons, the disruption of neuronal support by SCs and blood vessels contributes to neuropathy. Indeed, one notable pathological aspect of diabetic neuropathy is nerve degeneration as a consequence of ischemia and hypoxia. However, it remains unclear if the etiology of diabetic neuropathy occurs primarily due to neurovascular dysregulation or dysfunctional SCs since SCs and myelin are significantly affected by hyperglycemia in diabetic study models. Therefore, SCs might play a role in the pathogenic mechanism of diabetic neuropathy, possibly due to dysregulation of intracellular metabolic pathways, and can also be considered as potential targets for therapies.

Streptozotocin-treated diabetic rat or mice is widely used to induce hyperglycemia and insulin deficiency and/or resistance-producing *in vivo* models of diabetes. The induction of a diabetic state in rodents is dependent on the dose of streptozotocin and other aspects (Furman, 2021). Rodents treated with low doses (Wu and Huan, 2007) or a single high dose (Like and Rossini, 1976) of streptozotocin resembles type I diabetes mellitus while moderated doses of streptozotocin treatment by prior consumption of a high-fat diet (Reed et al., 2000) or streptozotocin treatment along with nicotinamide administration (Masiello et al., 1998) resembles type II diabetes. In a mouse model of streptozotocin-induced type I diabetes, hyperglycemia led to a decrease of caveolin-1 in SCs, which was reversed by insulin therapy. The authors showed that caveolin-1 downregulation in SCs leads to changes in the ratio of caveolin-1 to ErbB2 with altered neurotropism enhancing the response of SCs to neuregulins (Tan et al., 2003).

Using the diabetic db/db mouse, which carries a mutation in the leptin receptor gene inducing diabetes type II, Jia et al. (2018) demonstrated that administration of exosomes from high glucose stimulated SCs in the sciatic nerve caused peripheral neuropathy characterized by axonal damage. After finding high levels of miRs-28, −31a, and −130 in exosomes of high glucose-stimulated SCs, and a reduction of axonal growth after their *in vitro* administration, the authors showed *in vivo* that these exosomes and their putative target proteins are involved in diabetic peripheral neuropathy.

Exosome-based therapy has been considered an alternative potential tool for improving nerve regeneration in neuropathic diseases and following injury. For example, mesenchymal stem cell exosomes have been shown to promote axonal and vascular regeneration and immunomodulatory effects (Dong et al., 2019). Indeed, exosomes may play their beneficial roles by several means. For example, one of the beneficial mechanisms of mesenchymal stem cell exosomes for improving nerve degeneration is through promoting SC dedifferentiation and switching to a repair phenotype (Mao et al., 2019). Fan et al. (2021) treated diabetic db/db mice with engineered mesenchymal stem cells-exosomes loaded with miR146a. The authors found that the treatment improved neurovascular function as well as produced immunosuppressive effects, ultimately improving nerve regeneration. It might also be interesting to investigate the use of exosomes of human induced pluripotent stem cells-derived SCs as a putative therapeutic strategy for improving nerve regeneration following trauma or disease onset.

Disruption of mitochondrial calcium homeostasis is one of the hallmarks of neurodegeneration. Mitochondrial calcium release and leakage in SCs occur in injured and diabetic mouse nerves, respectively. This process is mediated by voltage-dependent anion channel 1 and triggers signaling pathways that activate the demyelination program. The use of both genetic and pharmacological therapeutic strategies for silencing and inhibiting voltage-dependent anion channel 1, respectively, reduced mitochondrial calcium release and prevented demyelination in rodent models of diabetic neuropathy and Charcot–Marie–Tooth disease (Gonzalez et al., 2017).

Demyelination is an early event in the pathomechanism of diabetic neuropathy. Consistent with these studies, mitochondria enlargement, disruption of normal cristae structure, and significant apoptosis is found in human SCs from biopsies of patients with diabetic neuropathy (Vincent et al., 2002). The authors showed similar morphological features in mice treated with 50% dextrose, which displayed acute hyperglycemia. The diabetic animals presented an increase in caspase-3 cleavage immunoreactivity in dorsal root ganglion and, to a lesser extent, in SCs, and an increase in reactive oxygen species in both cell types. The apoptosis found was a result of excess reactive oxygen species in the mitochondria. Adenovirus-mediated uncoupling protein 1 expression in dorsal root ganglion *in vitro* prevented glucose-induced neuronal cell death. Chinese herbal medicine has been proposed as a tool for preventing and treating diabetic peripheral neuropathy by inhibiting apoptosis of SCs under high-glucose conditions (Wang et al., 2023).

Chinese herbal medicine suppresses apoptosis is by reducing endoplasmic reticulum stress, oxidative stress, and the generation of advanced glycation end products. However, further *in vivo* experiments are needed to confirm the outcomes, mostly observed in *in vitro* studies. Another study using streptozotocin-treated diabetic rats showed that transplantation of SCs overexpressing nuclear factors erythroid 2-related factor 2, which plays a role in apoptosis and oxidative stress, restored nerve conduction velocity, myelin thickness, and vascularity of vasa nervorum (Tang et al., 2018).

Treatments targeting SCs with a focus on the regulation of intracellular metabolic pathways, supporting survival, mitochondrial calcium homeostasis, and metabolic-related membrane protein expression, after nerve trauma or neuropathy disease might directly influence nerve regeneration and carry therapeutic potential.

#### 3.2.3. Guillain–Barré syndrome

Guillain–Barré syndrome is an acute idiopathic autoimmune demyelinating disease characterized by inflammatory lesions in the peripheral nervous system. This neuropathy causes muscle weakness and paralysis but can also cause autonomic dysfunction. It is usually preceded by viral or bacterial infections and, besides demyelination, can result in primary and secondary axonal injury. T cells, B cells, macrophages, and complement system attacks gangliosides of axons and SCs. However, the pathogenesis of Guillain–Barré syndrome is not completely understood.

It is known that lipo-oligosaccharides of *Campylobacter jejuni* play a role in autoimmune neuropathies by mimicking human gangliosides found in the peripheral nervous system. The lipo-oligosaccharides of Campylobacter trigger the production of antibodies against host gangliosides, leading to Guillain–Barré syndrome (Moran et al., 2005).

Animal models of Guillain–Barré syndrome have been developed for studying complex cell interactions in this disease. Chickens fed with *Campylobacter jejuni* developed paralytic neuropathy, but the pathology ranged from no detectable changes to severe Wallerian-like degeneration (Li et al., 1996) Sensitization of rabbits with *Campylobacter jejuni* and GM1 ganglioside is another *in vivo* alternative model for studying the molecular pathogenesis of the disease (Shahrizaila and Yuki, 2011). A transgenic mouse model of constitutive expression of the co-stimulator B7.2/CD86 on antigen-presenting cells of the nervous system, named L31, resembles clinical and pathological features observed in patients with Guillain–Barré syndrome (Zehntner et al., 2003). When CD4^+^ T cells are depleted in L31 mice, the onset of the disease is accelerated and its prevalence is increased (Yang et al., 2014). For modeling both axonal and demyelinating variants in Guillain–Barré syndrome McGonigal et al. (2022) generated the glycosyltransferase-disrupted transgenic mice expressing GM1 ganglioside in neurons or SCs. This transgenic mouse model allows the auto anti-GM1 antibodies expressed to exclusively target GM1 in either SC or axonal membranes (Goodfellow and Willison, 2016). Interestingly, perisynaptic SCs, rather than inflammatory cells, are the main phagocytic cells to clear axonal debris in mouse models of acute motor axonal neuropathy (Cunningham et al., 2020).

The main available treatments for Guillain–Barré syndrome are immunotherapy-based plasmapheresis and intravenous immunoglobulins. However, some patients do not respond to the therapy, or the outcomes are suboptimal (Liu et al., 2018). Patients with Guillain–Barré syndrome present circulating immunoglobulin G autoantibodies against myelin but also against proliferating, non-myelinating human SCs. Sera from patients with Guillain–Barré syndrome recognized nerve-growth-cone associated proteins and epitopes involved in SC-axon interaction in distal tips of SCs process *in vitro* (Kwa et al., 2003). Guillain–Barré syndrome is very complex, and treatments might depend on the severity and age but most importantly on the variant and immune cell phenotypes and epitopes involved. Therefore, strategies targeting proteins and epitopes not only involved in SCs or axonal homeostasis but also that take a direct part in SC-axon interaction might be considered for treating Guillain–Barré syndrome.

### 3.3. Modeling peripheral injuries *in vivo*

#### 3.3.1. Potential therapeutic agents for peripheral nerve injuries

There is a high prevalence in peripheral nerve injuries (PNI) and other neuropathies in the PNS, resulting in life-long loss, dysfunction in several organs, and paralysis. Although microsurgical therapy is the gold standard treatment, other therapeutic approaches can improve nerve regeneration including biomaterials, gene therapy, and small molecules. Rodent, sheep, and pig animal models are used in preclinical studies after completing *in vitro* studies.

SCs have a crucial role in repairing injury and axonal regeneration in PNS. The finding of compounds promoting SC activation might improve peripheral nerve repair by using them as potential therapeutic agents. In the last decade, the regeneration potential of a number of molecular and gene agents has been evaluated in the mouse sciatic nerve injury model. They have shown potential applications in preclinical studies (Al-Saedi et al., 2023; Elhessy et al., 2023; Ferdowsi et al., 2023).

Several animal models mimic the PNI after nerve crushing or transection allowing the use of them as an effective model to study regeneration. Potential therapeutic agents were used to study the impact of this process. These therapeutic agents can be classified into different groups such as (1) anti-inflammatory agents, (2) neurotrophic factors and neuroactive cytokines (3) hormones and antioxidants, (4) chemotherapy agents, (5) transgenes, (6) extracellular vesicles, and (7) biomaterials which can recapitulate the characteristic of peripheral environment. These groups of potential therapeutic agents can be used as a neuroprotective treatment or effective treatment in nerve regeneration (Table 4).

**Table 4:** Potential drug or gene therapy approaches for peripheral nerve repair and axonal growth.

Animal model	Type of injury	Drug/gene delivery	Target evaluated	Major findings	Reference
Rat	PNI—SN crush	Cerebrolysin, dexamethasone, ascorbic acid	Neurons in SN	Improvement of behavior and histopathological changes and SN function after ascorbic acid administration. Neuroprotective effect	Elhessy et al. (2023)
Wistar rat	PNI—SN crush	Azithromycin	Neurons in SN	A significant effect was found in motor and sensory function	Ferdowsi et al. (2023)
Wistar Rat	PNI—SN crush	Nimodipine	Neurons in SN	Improvement in motor and sensory functions. The number of myelinated fibers increased. Pro-inflammatory factors were reduced. Neuroprotective effect	Al-Saedi et al. (2023)
C57Bl/6 mice	PNI—SN crusch	CD200R1	SN	CD200R1 has a role in recruitment of myeloid cells in the regeneration process. CD200R1 is involved in inflammatory reactions after injury, and contributes to functional improvement	Pannunzio et al. (2022)
Balb-C mice	PNI SN crushed by laser microdissection	Thymoquinone (*Nigella sativa*)	Neurons from DRG	TQ showed neuroprotective potential *in vitro*	Üstün et al. (2018)
C57Bl/6 mice hESC, (HUES64)	The tibial nerve was crushed	Botulinum neurotoxin A	Motor neurons	Peripheral motor axon regeneration is improved in mice and neurite growth was observed after MN differentiation from hESC	Adler et al. (2022) Franz et al. (2018)
Wistar rat, SCs isolated from Wistar rat	Removed 10 mm of SN	VPA (valproic acid)	Neurons	SN regeneration, increase SC proliferation	Gomez-Sanchez et al. (2022)
Wistar rat	SN gap less than 10 mm	Polyhydroyal-kanotes	SN engraftment	Can successfully sustain cell proliferation and adhesion *in vitro* and nerve regeneration across a 10 mm median nerve defect *in vivo*	Lizarraga-Valderrama et al. (2021)
Neonatal, Sprague–Dawley (SD) rats	Rat model of SN transaction	Hydrogel, let-7a	SN engraftment	Let-7a antagomir promoted peripheral nerve regeneration and is a biosafety molecule for *in vivo* application	Chen et al. (2023)
Wistar rat	PNI—SN crush	adMSCs-exosomes	SN	The results showed a significant improvement in axon regeneration after sciatic nerve injury *in vivo*	Bucan et al. (2019)
P2–P3 SD rat sciatic nerves		Exosomes derived from repair SCs	SN	SC-derived exosomes to this phenotype that enhances neurite growth are dependent on the increased expression of miRNA-21	Liu et al. (2022)

The nerve regeneration *in vivo* can be evaluated by withdrawal reflex latency, electrophysiology, histomorphometric analysis including axon diameter and myelin thickness, gastrocnemius muscle mass ratio, recovery of foot morphology, and footprint pattern.

#### 3.3.2. Neurotrophic factors, antioxidants, and anti-inflammatory agents for treating PNI

PNI affects sensory, motor and autonomic nerve functions in PNS. After the injury, the inflammatory response plays an important role in regeneration. Macrophages are recruited to the injury site and besides phagocytosing myelin and axonal debris, they interact with SCs to promote axonal regeneration. Macrophages also participate in the dedifferentiation SCs during the repair process. After a traumatic PNI, the patients can present long-lasting disabilities. SCs can regenerate after injury, however, the success of the functional recovery depends on several factors such as the size of the gap between the proximal and distal stump, connective tissue sheath integrity, the age of the patients, among others. To improve repair strategies after traumatic PNI, different approaches have been investigated.

A recent study evaluated the effect of Cerebrolysin, the anti-inflammatory drug dexamethasone, and ascorbic acid, which is a potent antioxidant agent, following nerve crush injury. The administration was performed on 32 adult rats. After administration of the 3 compounds, motor and sensory neuron function were improved. However, Vitamin C presented the most significant improvement. In addition, it showed antiapoptotic effects due to BAX/BCL2 ratio being reduced in SN (Elhessy et al., 2023).

It was also shown that inflammatory modulation can be used as a therapeutic approach to improve nerve regeneration in patients with chronic inflammation. In this study, after severe nerve crush injury in Wistar rats, 40 animals were immediately treated with azithromycin, an anti-inflammatory agent, either 15 or 150 mg/Kg/day for a week. The results showed incremental nerve regeneration after 1 week of treatment. The authors showed that the experimental group upregulated NGF and BDNF expression during the treatment, which contributed to improving axon regeneration. They suggested that azithromycin modulated the inflammatory response to improve sensory and motor neuron function (Ferdowsi et al., 2023).

Another recent study suggested that administration of nimodipine, a calcium channel blocker, showed a neuroprotective effect after neuronal injury. In this study, nerve crush injury was performed in Wistar rats. Nimodipine was administered a week after PNI. The treatment increased the number of myelinated fibers as well as NFG, and BDNF expression. The findings showed that motor and sensory neuron functions increased after treatment. Nimodipine can both reduce apoptosis and promote axon regeneration likely by modulating the CREB signaling pathway and suppressing pro-inflammatory factors (Al-Saedi et al., 2023).

Novel potential therapeutic targets involved in neuroinflammatory modulation have been identified. They activate and inhibit immune receptors such as CD300f, TREM2, CD200R1, and Siglecs. The blockage of CD200R1 resulted in a reduction of macrophages and monocytes in the SN lesion, suggesting an important role in the recruitment of myeloid cells in nerve regeneration. Also, CD200R1 showed a role in the immune response by recruiting monocytes in PNS and its inhibition impaired nerve regeneration. However, CD200R1 is not directly associated with myelin remotion or remyelination (Pannunzio et al., 2022).

Thymoquinone (TQ) acts as an antioxidant, anti-inflammatory, anti-epileptic, anticonvulsant, neuroprotective, and anticarcinogenic agent. The therapeutic potential of this compound was tested in rat diabetic peripheral neuropathy and traumatic facial nerve paralysis models. Evidence shows that TQ can improve nerve conduction velocity and can participate in SC proliferation and survival. In addition, studies showed that TQ can reduce proinflammatory cytokines secretion by SCs and sciatic nerve. Recently, a study administered two doses of TQ, at 50 and 75 nM, in a PNI mouse model induced by laser microdissection. The results showed an increased survival of neurons isolated from DRG, increased SCs and fibroblast proliferation, and more extension of neurites *in vitro*, at 75 nM (Üstün et al., 2018).

Evidence suggested that botulinum neurotoxin A (BoNT/A) can improve nerve regeneration and functional recovery after PNI. Studies have shown that BoNT/A may induce activation or proliferation of SCs, mast cells, and macrophages, in addition to promoting angiogenesis and increasing blood flow (Franz et al., 2018). BoNT/A has been indicated as a potential translational pharmacological candidate to improve motor neuron regeneration after PNI. Recently, a study suggested that the administration in mice with tibial nerve injury or hESC based model of Botulinum toxin A with clinical grade formulation (BoTX) previous to nerve repair may improve motor axon regeneration. The study suggested that BoTX can be used as a preconditioning treatment. BoTX was injected in the triceps sural muscle of mice with 0.1, 0.25, or 0.5 units (U) and the tibial nerve above the knee was crushed. They showed that BoTX induced neuromuscular sprouting compared with SHAM, as well as increased axonal reinnervation. To support these results hESC based model was treated *in vitro* and the results showed an increase in neurite growth (Franz et al., 2018 Adler et al., 2022).

#### 3.3.3. Biomaterials, epigenetic modulators, and hydrogels

During regeneration, SCs can myelinate axons and give support to unmyelinated axons. Evidence shows that an epigenetic modulation is involved in the repair process due to SC gene expression changes during the reprogramming step and regeneration. In this context, histone acetyltransferases (HATs) or histone deacetylation by histone deacetylases (HDACs) can play an important role in nerve regeneration as a potential treatment after PNI. In addition, HDACs have shown an important role in myelination (Gomez-Sanchez et al., 2022). Autografts are commonly used to repair peripheral nerve injury. A study designed novel bioabsorbable conduits based on hydroxyapatite/poly D-L-lactic acid (PDLLA)/poly{(lactic acid)-co-[(glycolic acid)-alt-(L-lysine)]} with sustained release of valproic acid (VPA). VPA shows clinical therapeutic effects similar to neurotrophic factors. In this study, SCs were isolated from Wistar rats and the effect of 10 ug/mL VPA was evaluated *in vitro*. The findings showed increased metabolic activity and proliferation of SC. Then, a conduit able to release VPA was implanted after removing 10 mm-length in the sciatic nerve of Wistar rats. The engraftment showed an improvement in axonal nerve regeneration after 12 weeks of implantation (Wu et al., 2021).

Nerve guidance conduits (NGCs) are an alternative treatment to autologous nerve engraftment for promoting nerve regeneration *in vivo*. However, regeneration by NGCs is limited to short gaps of less than 10 mm. A study showed that a 75:25 poly(3-hydroxyoctanoate)/poly(3-hydroxybutyrate) blend (PHA-NGCs) can induce the proliferation of SC line RT4-D6P2T *in vitro*. After *in vivo* engraftment in a Wistar rat for 12 weeks, the results showed an improvement in nerve regeneration. Also, the density and number of SCs and axon diameter were increased in the sciatic nerve. The study suggested that (PHA-NGCs) can be used as an attractive option to nerve regeneration in translational medicine (Lizarraga-Valderrama et al., 2021).

Tissue-engineered nerve grafts have been established as a prospective alternative for regenerative medicine. They can also be combined with cells or small molecules. Recent studies have reported several microRNAs (miRNAs) able to regulate axon regeneration after PNI. Let-7 is a miRNA involved in different biological processes including regulation of NGF secretion from SCs, increased SCs proliferation and migration *in vitro*, and axon outgrowth promotion *in vivo*. In a recent study, a hydrogel supported the release of let-7a antagomir. This approach consisted of adding let-7a antagomir into a chitosan conduit to construct a chitosan hydrogel scaffold tissue-engineered nerve graft and its implantation in a rat model of sciatic nerve transection. Let-7a antagomir at 5 nmol was injected into the conduit mixed with hydrogel and transplanted to challenge a nerve 7 mm-gap. Let-7a improved nerve regeneration and functional recovery and showed biosafety as a molecule for *in vivo* application (Chen et al., 2023).

#### 3.3.4. SCs-derived exosomes as potential therapy

Recently, extracellular vesicles (EVs) have been used as an alternative approach for the delivery of specific cargo. EVs are derived from different types of cells and mediate intracellular communication among cells. Thus, EVs can load several molecular cargoes such as RNA, DNA, and protein. The size capacity of molecular cargo in EVs is high. For example, they can deliver a full-length protein compared with other types of delivery systems. Exosomes have a crucial function in cell–cell communication and they can improve nerve regeneration. Evidence shows that SCs-derived exosomes play a role in protecting the spinal cord after injury (Huang et al., 2023).

Therefore, EVs derived from cells able to overexpress and deliver candidate genes have been used as a powerful tool to generate and deliver engineer EVs (eEVs) to specific cells. Some protocols have used EVs derived from rat SCs to deliver cargo able to mediate regeneration (Xia et al., 2020). Other studies focused on using EVs derived from SCs as potential approaches for treatments and biomarkers (López-Leal et al., 2020; Huang et al., 2023).

A study evaluated the regeneration potential of exosomes isolated from adipose-derived mesenchymal stem cells (adMSCs) from Wistar rat. The capacity of exosomes to promote neurite growth was confirmed *in vitro* after DRG-adMSCs co-culture. Also, adMSC exosomes were directly injected in the proximal and distal stumps of sciatic nerve after lesion. After 21 days, functional recovery was observed by evaluating the walking behavior of rats’ footprint (Bucan et al., 2019).

#### 3.3.5. Advantages and limitations of *in vivo* models

*In vivo* models of peripheral neuropathies allow the study of different aspects of the disease in terms of cellular interactions in the disturbed microenvironment. However, animal models have some limitations, including the lack of recapitulation of human disease. In addition, most translational clinical studies fail to achieve the level of positive outcomes observed in animals because of inter-species differences.

Neurological diseases carry pathophysiologic heterogeneity and biological complexity, so individual differences should be considered for their diagnosis and treatment. Precision medicine is a medical approach that takes into account accurate personal genomic and proteomic analysis to guide treatment that may work best for the patient. In addition, the use of patient-derived induced pluripotent stem cells opens up the possibility for *in vitro* drug screening considering the personal response to each drug tested. However, this *in vitro* model also has its own limitations, including the lack of complex multicellular interactions. For instance, to date, there is no efficient human myelinating co-culture model of peripheral neuropathies, in particular for studying demyelinating neuropathies. Improving protocols for generating functional human SCs with the specific desired phenotype *in vitro* may help produce precise diagnoses and treatments for individual patients.

## 4. Future perspectives

The study of SCs and axon interaction is crucial to understand nerve regeneration mechanisms and to search for new treatments. During the last few years, a large number of novel agents were studied as potential treatments to move to clinical translation. Although several studies seem promising, there are limitations to evaluate the novel targets, including efficiency and consistency of *in vitro* and *in vivo* protocols. Thus, the use of iSCs derived from patients with optimized protocols is a useful tool to model neuropathies disease and perform drug screening *in vitro*.

## 5. Conclusion

This review summarizes the most relevant studies published so far on the use of drugs to target SCs in diseases affecting peripheral nerves. The cellular and molecular mechanisms of diseases such as Charcot–Marie–Tooth, diabetic neuropathy, and Guillain–Barré, in addition to their progression, are associated with different factors involving microenvironment, gene expression signatures, among others. The direct interaction of SCs with axons or with factors released by the nerve environment such as growth factors, neurotrophic factors, cytokines, and exosomes act as a defense response mechanism and is crucial for repairing the nerve. Therefore, the regulation of factors released by SCs may be the key to improving the regeneration. Despite several preclinical studies that have shown potential to move to clinics for treating peripheral nerve diseases and injuries, more evidence is needed to demonstrate the efficacy of treatments that target SCs in terms of nerve functionality and reinnervation.

## Author contributions

JTO, CEGL, NW, and CY wrote the manuscript. JTO and CEGL compiled the figures. All authors contributed to the article and approved the submitted version.
